# Solvent-Dependent Efficacy of *Moringa Oleifera* Leaf Extracts on Methicillin-Resistant *Staphylococcus Aureus*: Assessments on Antibacterial and Antioxidant Properties

**DOI:** 10.21315/tlsr2026.37.1.13

**Published:** 2026-03-31

**Authors:** Ilakhiya Paavai Arunasalam, Seri Narti Edayu Sarchio, Nur Liyana Daud, Suhaili Shamsi, Mohd Nasir Mohd Desa, Muhamad Hafiz Abd Rahim

**Affiliations:** 1 Department of Biomedical Science, Faculty of Medicine and Health Sciences, Universiti Putra Malaysia, 43400 UPM Serdang, Selangor, Malaysia; 2 Department of Biochemistry, Faculty of Biotechnology and Biomolecular Sciences, Universiti Putra Malaysia, 43400 UPM Serdang, Selangor, Malaysia; 3 Department of Food Science, Faculty of Food Science and Technology, Universiti Putra Malaysia, 43400 UPM Serdang, Selangor, Malaysia

**Keywords:** *Moringa oleifera*, Solvent Extraction, Methicillin-resistant *Staphylococcus aureus*, Antibacterial, Antioxidant, Moringa oleifera, Pengekstrakan Pelarut, Methicillin-resistant Staphylococcus aureus, Antibakteria, Antioksidan

## Abstract

The global surge in antibiotic resistance, particularly methicillin-resistant *Staphylococcus aureus* (MRSA), presents a significant public health challenge and emphasises the necessity for alternative therapeutic strategies. *Moringa oleifera* is rich in bioactive phytochemicals with established antimicrobial properties, however, the impact of various extraction solvents on its efficacy against MRSA has not been thoroughly investigated. This study offers a direct comparison of ethanolic, methanolic and aqueous *M. oleifera* leaf extracts (MOLE) to identify the optimal solvent for enhancing antibacterial and antioxidant activities. MOLE was extracted through maceration, and antibacterial activity against MRSA was assessed using colony-forming unit (CFU) assays at concentrations of 50 mg/mL, 100 mg/mL and 200 mg/mL, with vancomycin serving as the positive control. Antioxidant capacity was quantified using the ferric reducing antioxidant power (FRAP) assay, while phytochemical profiles characterised via liquid chromatography–mass spectrometry (LC–MS). The ethanolic extract exhibited the highest bioactivity, achieving complete MRSA inhibition at 100 mg/mL and demonstrating efficacy statistically comparable to vancomycin at 50 mg/mL. It also showed significantly greater antioxidant capacity across all tested concentrations. LC–MS analysis linked this enhanced activity to a richer composition of flavonoids and phenolic acids in the ethanolic extract. In summary, ethanol emerged as the most effective solvent for extracting antibacterial and antioxidant compounds from *M. oleifera* leaves. The findings underscore the potential of ethanolic MOLE as a natural therapeutic candidate against MRSA, warranting further investigation in preclinical models.

## Abstrak

Peningkatan global dalam rintangan antibiotik, khususnya methicillin-resistant Staphylococcus aureus (MRSA), merupakan cabaran besar kepada kesihatan awam dan menekankan keperluan kepada strategi terapeutik alternatif. Moringa oleifera kaya dengan fitokimia bioaktif yang terbukti mempunyai sifat antimikrob, namun kesan pelbagai pelarut pengekstrakan terhadap keberkesanannya melawan MRSA masih belum dikaji secara menyeluruh. Kajian ini menawarkan perbandingan langsung antara ekstrak daun M. oleifera (MOLE) secara etanolik, metanolik, dan akueus untuk mengenal pasti pelarut yang paling optimum dalam meningkatkan aktiviti antibakteria dan antioksidan. MOLE diekstrak melalui proses maserasi, dan aktiviti antibakteria terhadap MRSA dinilai menggunakan ujian unit pembentuk koloni (colony-forming unit, CFU) pada kepekatan 50 mg/mL, 100 mg/mL dan 200 mg/mL, dengan vancomycin digunakan sebagai kawalan positif. Keupayaan antioksidan diukur menggunakan ujian ferric reducing antioxidant power (FRAP), manakala profil fitokimia dicirikan melalui kromatografi cecair–jisim spektrometri (liquid chromatography–mass spectrometry, LC–MS). Ekstrak etanolik menunjukkan bioaktiviti tertinggi, dengan pencapaian perencatan MRSA sepenuhnya pada kepekatan 100 mg/mL dan menunjukkan keberkesanan yang secara statistik setara dengan vancomycin pada kepekatan 50 mg/mL. Ia juga menunjukkan keupayaan antioksidan yang jauh lebih tinggi pada semua kepekatan yang dikaji. Analisis LC–MS mengaitkan peningkatan aktiviti ini dengan komposisi fitokimia yang lebih kaya, menjadikan MOLE etanolik calon terapeutik semula jadi yang berpotensi terhadap MRSA dan memerlukan kajian lanjut dalam model praklinikal.

**HIGHLIGHTS**

- Ethanolic extract of *Moringa oleifera* leaf showed the highest antibacterial activity, achieving complete inhibition of methicillin-resistant *Staphylococcus aureus* (MRSA) at 100 mg/mL and demonstrating efficacy statistically comparable to vancomycin at a concentration of 50 mg/mL.
- Ethanol was identified as the most effective solvent for extracting antioxidant compounds, with the ethanolic extract consistently showing the highest ferric reducing antioxidant power (FRAP) values across all tested concentrations (50 mg/mL, 100 mg/mL and 200 mg/mL).
- LC-MS analysis confirmed that the superior bioactivity of the ethanolic extract is linked to a more diverse phytochemical profile, rich in flavonoids (like quercetin-3-galactoside and kaempferol-7-O-glucoside) and phenolic acids.

## INTRODUCTION

Antimicrobial drugs are essential in the management of infectious diseases, however, their extensive and frequently inappropriate use has expedited the emergence of antimicrobial resistance (AMR), now acknowledged as a significant global health threat. AMR is associated with increased morbidity, mortality and healthcare costs, with an estimated 1.27 million deaths directly attributed to resistant infections in 2019 (Naghavi *et al*. 2024). In the absence of effective interventions, AMR-related deaths are projected to escalate to 10 million annually by 2050 (Murray *et al*. 2022).

Among antibiotic-resistant pathogens, *Staphylococcus aureus*, particularly methicillin-resistant *S. aureus* (MRSA), presents a persistent clinical challenge due to its virulence, adaptability and limited therapeutic options. The *mecA* gene, which encodes the low-affinity penicillin-binding protein 2a (PBP2a), confers resistance to nearly all β-lactam antibiotics (Turner *et al*. 2019). MRSA prevalence remains substantial worldwide, with 35% methicillin resistance reported among *S. aureus* isolates (World Health Organization 2022). In Malaysia, reported rates range from 17.2% to 28.1% (Hamasalih *et al*. 2022), underscoring the urgency of identifying alternative treatment strategies. Natural products, particularly plant-derived antimicrobials, have garnered increasing attention as potential adjuncts or alternatives to conventional antibiotics due to their chemical diversity and lower risk of adverse effects. *Moringa oleifera*, known as ‘Kelor’ in Malay and commonly referred to as the drumstick tree, is a well-established medicinal plant with documented antimicrobial, antioxidant, anti-inflammatory and anticancer properties (El-Sherbiny *et al*. 2024; Akinduti *et al*. 2022). Its leaves contain flavonoids, tannins and alkaloids that have been reported to disrupt bacterial membranes, inhibit enzymatic pathways and neutralise reactive oxygen species, mechanisms that may collectively contribute to its antibacterial activity (Mehmood *et al*. 2022; Aboud *et al*. 2023). Despite its proven potential, the antimicrobial efficacy of *M. oleifera* leaf extract (MOLE) is highly dependent on the extraction method, particularly the solvent used. The polarity of the solvent greatly affects the solubility and yield of phytochemicals, which in turn affects antibacterial and antioxidant activity (Deepali *et al*. 2025; Othman *et al*. 2021). Despite its therapeutic potential, comparative data on how different solvents affect the efficacy of MOLE against MRSA remain limited.

To address this gap, the present study evaluated the influence of three commonly used solvents, ethanol, methanol and aqueous, on the phytochemical profile, antioxidant capacity and antibacterial activity of MOLE against MRSA. We hypothesised that the ethanolic extract would exhibit superior antibacterial and antioxidant properties due to ethanol’s ability to solubilise a broader spectrum of bioactive phytochemicals. By integrating colony-forming unit (CFU) assays with antioxidant evaluation and LC–MS phytochemical profiling, this study aims to identify the extraction method that maximises the therapeutic potential of MOLE. The findings provide a foundation for developing plant-based antimicrobial and antioxidative agents with relevance for managing MRSA and other multidrug-resistant pathogens.

## MATERIALS AND METHODS

### Sample Preparation and Extraction

A total of 250 g of *M. oleifera* leaves was collected from Selangor and Johor, Malaysia, and their taxonomy was verified at the Biodiversity Unit, Institute of Bioscience, Universiti Putra Malaysia (voucher specimen: KM 0197/25). The leaves were thoroughly washed, air-dried at room temperature for three days, and then oven-dried at 50°C for an additional three days until a constant weight was achieved. The dried leaves were ground into a fine powder using a high-pressure grinder (Rong Tsong, Taiwan), resulting in 64.62 g of leaf powder, which was stored in airtight containers at room temperature until required. Extraction was performed by maceration using 80% ethanol, 80% methanol and distilled water (aqueous). For each solvent, the powdered samples were soaked for 72 h at room temperature with intermittent shaking. The resulting suspensions were filtered, and the ethanol and methanol extracts were concentrated under reduced pressure using a rotary evaporator at 50°C. The aqueous extract was reduced to one-third of its volume on a hot plate at 50°C before freeze-drying. All extracts were lyophilised to obtain a dried powder form of MOLE, weighed to determine the extraction yield gravimetrically, and stored at 4°C until further analysis (Zahid *et al*. 2025).

### Antibacterial Assay

#### Media preparation

Mueller–Hinton Agar (MHA), Mueller–Hinton Broth (MHB) and Tryptone Soy Agar (TSA) were obtained from HiMedia (India) and prepared according to the manufacturer’s instructions.

#### Bacterial strain

The bacterial strain utilised in this study was methicillin-resistant *S. aureus* (MRSA; ATCC 700699). The strain was maintained at −30°C in Brain Heart Infusion (BHI) broth supplemented with 20% glycerol and sub-cultured on TSA prior to use.

#### Preparation of bacterial inoculum

To maintain bacterial viability, MRSA was sub-cultured on TSA and incubated overnight at 37°C under aerobic conditions. Subsequently, two isolated colonies were inoculated into 10 mL of Mueller-Hinton Broth (MHB) and incubated at 37*°*C for 24 h. The culture was then adjusted to a 0.5 McFarland standard (≈1 × 10^8^ CFU/mL) using sterile saline.

#### Colony-forming unit (CFU) assay

MOLE extracts were prepared by reconstituting the lyophilised MOLE powders in distilled water to final concentrations of 50 mg/mL, 100 mg/mL and 200 mg/mL. For each treatment group, 5 μL of the adjusted MRSA suspension was introduced into 5 mL of MHB containing the respective MOLE extract, resulting in a final bacterial concentration of approximately 1 × 10^5^ CFU/mL. Control groups included a negative control (bacteria in MHB only) and a positive control (bacteria in MHB with vancomycin at 30 μg/mL). The concentration of vancomycin (30 μg/mL) was selected based on concentrations frequently employed in studies investigating MRSA inhibition, in accordance with the susceptibility testing standards set by the Clinical and Laboratory Standards Institute (CLSI 2024). All experimental and control groups were incubated at 37°C in a shaking incubator for 24 h. Following incubation, each mixture was serially diluted 10-fold, and 100 μL of the diluted suspension was evenly spread onto the surface of the MHA plates using a sterile cotton swab to ensure uniform bacterial growth. The plates were incubated at 37°C for 18–24 h. Colony counts were conducted the following day, CFU/mL values were calculated (Shamsi *et al*. 2022), and the percentage inhibition of bacterial growth was determined using formula (1):


(1) $$\text{Percentage inhibition }(\%)=\frac{\left({\text{CFU}}_{\text{control}}-{\text{CFU}}_{\text{treated}}\right)}{{\text{CFU}}_{\text{control}}}\times 100$$

#### Ferric reducing antioxidant power (FRAP) assay

**FIGURE 1:**
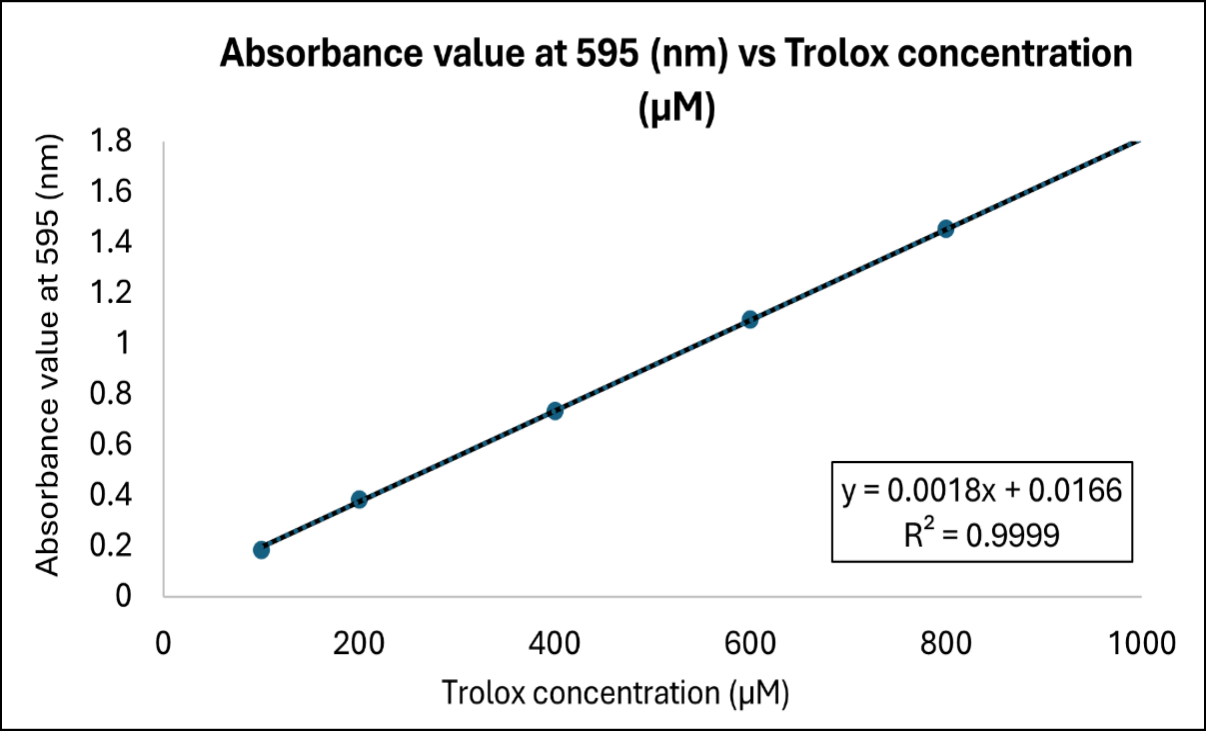
Standard curve of Trolox concentrations at 100 to 1,000 μM.

The ferric reducing antioxidant power (FRAP) assay was conducted according to the method of Clarke *et al*. (2013), with slight modifications. The FRAP reagent was freshly prepared by mixing 300 mM acetate buffer (pH 3.6), 10 mM 2,4,6-tripyridyl-s-triazine (TPTZ) in 40 mM HCl, and 20 mM FeCl_3_·6H_2_O in a 10:1:1 ratio, followed by pre-incubation at 37°C. Three solvent-based extracts of MOLE (ethanol, methanol and aqueous) were prepared at concentrations of 50 mg/mL, 100 mg/mL and 200 mg/mL. Prior to the analysis, all samples were diluted 50-fold to ensure that the absorbance readings were within the linear range of the assay. For each reaction, 20 μL of diluted MOLE was mixed with 180 μL of FRAP reagent in a 96-well plate. The plates were then incubated in the dark at room temperature for 30 min. Following incubation, the absorbance was measured at 595 nm using a microplate reader (TECAN, Infinite F50, Austria). Trolox served as the antioxidant standard, and standard solutions were prepared at concentrations of 100, 200, 400, 600, 800 and 1,000 μM to construct the standard curve (Fig. 1). All measurements were performed in triplicate. The results were expressed as μmol ferric equivalent (FE) per gram of extract.

### Phytochemical Identification Using Liquid Chromatography Analysis

Phytochemical profiling of MOLE was performed using liquid chromatography–tandem mass spectrometry (LC-MS/MS). Instrument performance was validated using an ESI tuning mix prior to analysis, ensuring mass accuracy was maintained within ±5 ppm for all detected ions. Each extract (ethanolic, methanolic and aqueous) was reconstituted at a concentration of 1 mg/mL prior to analysis. Analytical separation and detection were performed using an Agilent 1290 Infinity LC system coupled with an Agilent 6520 Accurate-Mass Q-TOF mass spectrometer equipped with a dual electrospray ionisation (ESI) source (Agilent Technologies Inc., California, USA). The ethanolic and aqueous extracts were analysed in positive ESI mode, whereas the methanolic extract was analysed in negative ESI mode to optimise the ionisation efficiency based on compound polarity. Mass spectra were acquired over the m/z range of 100–1,000 for the positive ion mode and 115–1,000 for the negative ion mode. Compound identification and profiling were performed using the Agilent MassHunter Qualitative Analysis software based on accurate mass measurements, isotopic pattern matching, and fragmentation profiles. The identified compounds were tentatively annotated by comparison with the published literature and database references (Zahid *et al*. 2025).

### Statistical Analysis

All statistical analyses were performed using GraphPad Prism software (GraphPad Software Inc., Version 10.4.2, San Diego, CA, USA). To assess the antibacterial efficacy of MOLE against MRSA, one-way analysis of variance (ANOVA) was conducted to compare the effects of varying MOLE concentrations (50 mg/mL, 100 mg/mL and 200 mg/mL) with those of vancomycin (30 μg/mL). For antioxidant analysis, two-way ANOVA was employed to evaluate the impact of the extraction solvent (ethanol, methanol and aqueous) and concentration on FRAP values. Post-hoc comparisons were conducted where appropriate. Data are expressed as mean ± standard error of the mean (SEM) from triplicate measurements unless otherwise indicated. A *p*-value less than 0.05 (*p* < 0.05) was considered statistically significant.

## RESULTS

### Antibacterial Activity of MOLE

**FIGURE 2:**
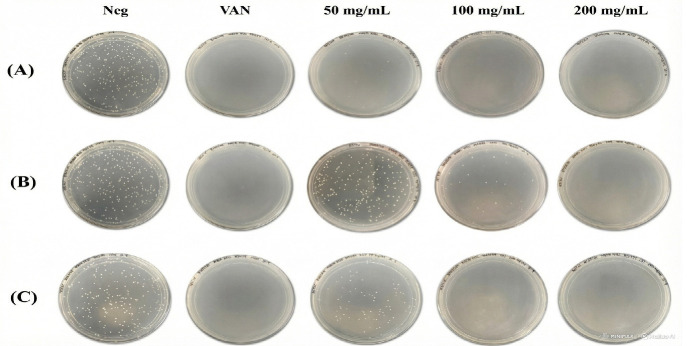
Representative images of MRSA cultured on Mueller-Hinton agar 24 hours after exposure to (A) ethanolic, (B) methanolic, and (C) aqueous MOLE extracts at varying concentrations (50 mg/mL, 100 mg/mL and 200 mg/mL). Untreated culture served as the negative control (Neg), while vancomycin (VAN, 30 μg/mL) was used as the positive control.

The antibacterial activity of *M. oleifera* leaf extract (MOLE) against methicillin-resistant *S. aureus* (MRSA) was assessed using a colony-forming unit (CFU) assay following a 24-hour incubation period. Three distinct extract types (ethanolic, methanolic, and aqueous) were evaluated at concentrations of 50 mg/mL, 100 mg/mL and 200 mg/mL. Vancomycin (30 μg/mL) was used as the positive control (Fig. 2).

**FIGURE 3:**
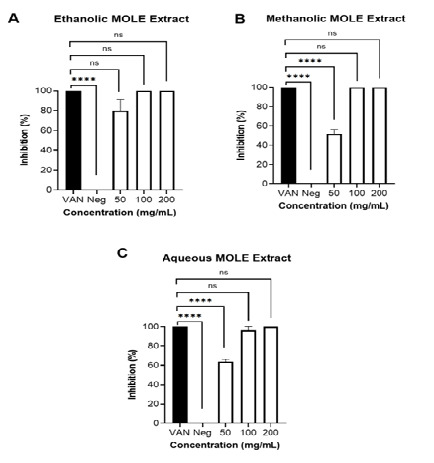
Percentage inhibition of MRSA following exposure to (A) ethanolic, (B) methanolic, and (C) aqueous MOLE extracts at different concentrations (50 mg/mL, 100 mg/mL and 200 mg/mL) over 24 h. Bacteria in Mueller-Hinton broth only were used as the negative control (Neg), while vancomycin (VAN, 30 μg/mL) served as the positive control. Data are presented as mean ± SEM (*n* = 3–4). Statistical significance was determined using one-way ANOVA followed by Dunnett’s post-hoc test. Significant differences to the positive control are denoted by **** (*p* < 0.0001), and ‘ns’ denotes no significant difference.

The ethanolic MOLE demonstrated the most potent antibacterial activity across all tested concentrations (Fig. 3A). Complete inhibition of MRSA growth was observed at both 100 mg/mL and 200 mg/mL, with inhibition levels statistically comparable to vancomycin (*p* > 0.05). At 50 mg/mL, the ethanolic extract still inhibited approximately 84% of bacterial growth, showing no significant difference from the positive control. This consistent efficacy across concentrations underscores ethanol as the most effective solvent for extracting antibacterial constituents from *M. oleifera* leaves.

The methanolic extract also demonstrated notable antibacterial activity (Fig. 3B), achieving complete inhibition at 100 mg/mL and 200 mg/mL. However, at 50 mg/mL, inhibition was decreased to approximately 60%, which was significantly lower than vancomycin (*p* < 0.0001). These results indicate a stronger dependence on extract concentration compared with the ethanolic extract.

The aqueous extract displayed moderate antibacterial effects (Fig. 3C). At 50 mg/mL, inhibition reached approximately 64%, significantly lower than vancomycin (*p* < 0.0001). At 100 mg/mL, the extract achieved near-complete inhibition (~96%), and complete inhibition was observed at 200 mg/mL, with activity comparable to the positive control (*p* > 0.05). Although less potent at lower concentrations, the aqueous extract retained measurable efficacy at higher doses.

Across all extracts, MRSA inhibition increased in a dose-dependent manner. The ethanolic extract was the most effective at all concentrations, followed by methanolic and aqueous extracts. These findings underscore the importance of solvent selection in maximizing antibacterial potency.

### Antioxidant Activity of MOLE

**FIGURE 4:**
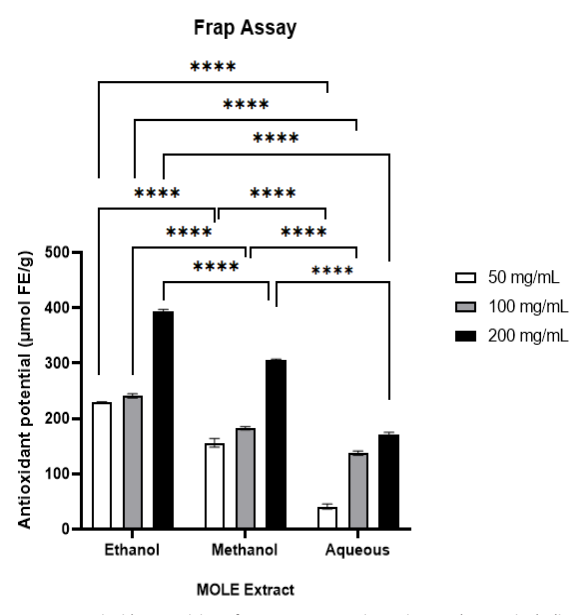
Antioxidant activity of MOLE extracts in various solvents, including ethanolic, methanolic, and aqueous at concentrations of 50 mg/mL, 100 mg/mL and 200 mg/mL, measured using the FRAP assay. Results are expressed as μmol ferric equivalent (FE) per gram of extract (μmol FE/g). Data are presented as mean ± SEM (*n* = 3). Statistical analysis was performed using two-way ANOVA, followed by Tukey’s post-hoc test to evaluate differences between extract types and concentrations. Significant differences are indicated by **** (*p* < 0.0001).

The ferric reducing antioxidant power (FRAP) assay was utilised to evaluate the antioxidant capacity of MOLE. Three solvent extracts (ethanolic, methanolic and aqueous) were assessed at concentrations of 50 mg/mL, 100 mg/mL and 200 mg/mL. All MOLE extracts demonstrated a concentration-dependent increase in antioxidant capacity (Fig. 4). Among the three, the ethanolic extract consistently showed the highest FRAP values, increasing from 229.67 μmol FE/g to 400.94 μmol FE/g at 200 mg/mL. The methanolic extract exhibited moderate reducing capacity, rising from 141.89 μmol FE/g to 307.33 μmol FE/g over the same concentration range. In contrast, the aqueous extract displayed the lowest antioxidant activity, with an increase from 32.83 μmol FE/g to 177.53 μmol FE/g across the same concentration range. These findings indicate that ethanol is the most efficient solvent for isolating redox-active phytochemicals, likely due to its capacity to solubilise a broader spectrum of phenolics and flavonoids.

### Phytochemical Analysis of MOLE

Liquid chromatography–mass spectrometry (LC-MS) was used to characterise the phytochemical constituents of the ethanolic, methanolic and aqueous extracts of MOLE. Given the different polarities of the extraction solvents, each extract was expected to exhibit a unique chemical composition. The data obtained were compared and validated using proprietary (METLIN) and online databases, such as PubChem, ScienceDirect and Nature.

LC–MS analysis revealed distinct phytochemical profiles across the three extracts (Table 1). As expected, solvent polarity influenced the diversity and abundance of detected compounds. Ethanolic MOLE contained the most complex phytochemical mixture, including flavonoid glycosides (e.g., quercetin-3-galactoside, kaempferol-7-O-glucoside) and phenolic acids (e.g., gallic acid). Methanolic MOLE was rich in phenolic acids and simple flavonoids, while the aqueous extract predominantly contained hydrophilic compounds such as gallic acid. These differences align with the functional results, suggesting that the enhanced antibacterial and antioxidant activities of the ethanolic extract may be attributed to its broader spectrum of bioactive metabolites.

## DISCUSSION

The present study demonstrated that all three *M. oleifera* leaf extracts (MOLE), ethanolic, methanolic and aqueous, exhibited dose-dependent antibacterial activity against MRSA, with increasing efficacy alongside extract concentration. Both ethanolic and methanolic extracts achieved complete bacterial inhibition at 100 mg/mL, while the aqueous extract reached full inhibition at 200 mg/mL. The observed plateau between 100 mg/mL and 200 mg/mL suggested that 100 mg/mL may represent the minimum effective concentration under the tested conditions for ethanol and methanol extracts. Although water-based extraction is typically less efficient in solubilising hydrophobic bioactives, the complete inhibition observed at higher concentrations underscores the presence of effective water-soluble antimicrobial compounds. At the lowest concentration tested (50 mg/mL), the ethanolic extract exhibited antibacterial activity statistically comparable to vancomycin (30 μg/mL) and was significantly higher than the methanolic and aqueous extracts. This highlights that ethanol is the most effective solvent for extracting antibacterial constituents from MOLE. In contrast, both methanolic and aqueous extracts showed significantly lower inhibition at this concentration, indicating reduced efficacy and suggesting that higher doses are required to achieve comparable antibacterial performance. These findings are consistent with the known solvent selectivity of plant phytochemicals, where ethanol has been widely reported to extract a broad range of antimicrobial metabolites including moderately polar flavonoids and phenolic acids (Mehmood *et al*. 2022; Aboud *et al*. 2023).

The FRAP assay results further supported the solvent-dependent differences in extract potency, with all extracts showing a significant concentration-dependent increase in antioxidant capacity. The ethanolic extracts consistently exhibited the highest FRAP values across all concentrations, followed by methanolic and, the aqueous extracts. Statistical analysis confirmed significant interactions between the extract type and concentration. The high FRAP values of the ethanolic extract are consistent with the known ability of phenolics and flavonoids to reduce Fe^3+^ to Fe^2+^ ions, a hallmark of antioxidant activity (Baldisserotto *et al*. 2023). The aqueous extract, limited to highly polar constituents, such as ascorbic acid, displayed minimal antioxidant capacity, particularly at 50 mg/mL. This further supports the suitability of ethanol as the preferred solvent to maximise the antioxidant potential of MOLE.

LC-MS analysis offers further insights into the phytochemical foundation of the observed bioactivities. The methanolic extract contains abundant phenolic acids and flavonoids, including gallic acid, chlorogenic acid and hydrojuglone glucosides, which are known for their antimicrobial and antioxidant properties (Baldisserotto *et al*. 2023; El-Sherbiny *et al*. 2024; Deepali *et al*. 2025; Sang *et al*. 2024; Shamsi *et al*. 2022). The ethanolic extract exhibited the most diverse profile, encompassing flavonoid glycosides such as quercetin-3-galactoside, kaempferol-7-O-glucoside and saponarin, which are metabolites that are strongly linked to membrane disruption and inhibition of bacterial enzymes and nucleic acid synthesis (Oluewu *et al*. 2024; Khalid *et al*. 2023; Jing *et al*. 2022). Although the aqueous extract contained fewer diverse compounds, primarily gallic acid and chlorogenic acid, its bioactivity at higher concentrations suggested that even simple phenolics can impart antibacterial effects when present in sufficient quantities.

Correlating the phytochemical data with functional outcomes revealed that the superior antibacterial and antioxidant activities of the ethanolic extract are likely attributed to its higher content and broader spectrum of bioactive metabolites. These findings are consistent with earlier reports that highlight the effectiveness of ethanol in extracting medium-polarity compounds such as flavonoids and phenolic glycosides. These compounds exhibit antimicrobial properties through various mechanisms, including membrane disruption, oxidative stress induction and the inhibition of biofilm formation (Aboud *et al*. 2023; Nugraha *et al*. 2023). In contrast, the methanolic extract, although somewhat less diverse, still demonstrated strong bioactivity owing to its phenolic acid content (Othman *et al*. 2021), whereas the aqueous extract was least potent at lower concentrations but retained measurable activity at higher doses.

These findings highlight the critical role of solvent selection in phytochemical extraction, particularly for antimicrobial and antioxidant purposes. Ethanol has proven to be the most effective solvent for enriching MOLE with therapeutically relevant bioactive compounds. While the results offer strong evidence of the antibacterial and antioxidant potential of MOLE, several limitations need to be addressed. First, the study was confined to *in vitro* assays, which do not fully capture the complexity of *in vivo* systems, including host immune responses, tissue distribution and metabolic interactions. Second, the phytochemical analysis conducted is qualitative, identifying the presence of compounds without quantifying their concentrations. The LC–MS profiling was performed qualitatively due to the lack of available reference standards and limited access to quantitative calibration workflows. The instrument was configured for qualitative accurate-mass identification, which precluded the use of external standard calibration or internal standard normalisation. Consequently, the phytochemicals are reported based on relative ion abundance rather than absolute concentrations. This limitation hinders the establishment of quantitative correlations between the phytochemicals and the observed antibacterial or antioxidant activities. Future research should employ quantitative LC–MS/MS analysis with authenticated standards to ascertain the concentrations of key compounds such as quercetin glycosides, kaempferol derivatives, chlorogenic acid and gallic acid. Although the superior performance of the ethanolic extract is attributed to its richer phytochemical profile, it remains unclear whether this effect is due to higher quantities of specific compounds or synergistic interactions among them. Future studies should incorporate validated quantitative LC-MS/MS methods to measure these key phytochemicals, which are crucial for establishing structure-activity relationships and standardising the extract for potential clinical applications.

The mechanistic explanations proposed in this study such as membrane disruption, oxidative stress induction and interference with bacterial metabolic pathways are based on previously reported activities of flavonoids and phenolic acids but were not experimentally validated in the present study. No mechanistic assays, such as membrane permeability tests, ROS quantification, efflux pump inhibition studies or gene expression analysis, were performed. Therefore, the mechanisms discussed should be regarded as hypotheses that require verification through dedicated mechanistic studies. Future investigations should incorporate targeted mechanistic assays to elucidate the specific roles of key compounds, particularly quercetin- and kaempferol-based derivatives, in mediating MRSA inhibition and antioxidant activity. Additionally, evaluating the synergistic interactions between MOLE and clinically relevant antibiotics, particularly vancomycin, will be crucial for determining the potential of combination therapies. Studies assessing the release kinetics, physicochemical stability, and suitability of MOLE for incorporation into delivery systems such as hydrogels or wound dressings will further support its translational development.

Cytotoxicity assessments on mammalian cell lines are also necessary to evaluate the safety and therapeutic window of MOLE for potential biomedical applications. Another limitation concerns the potential variability in the phytochemical composition of *M. oleifera* leaves. Factors such as plant maturity, environmental conditions, harvesting season and post-harvest processing can significantly influence metabolite content and, consequently, the reproducibility of antibacterial and antioxidant activities. Variability may also arise during extraction, as small differences in solvent quality, drying parameters or extraction duration can affect yield and compound composition. These sources of variation may pose challenges for standardising MOLE in preparation for large-scale, translational or commercial applications.

## CONCLUSION

This study illustrates that the selection of the extraction solvent significantly influences the phytochemical composition and bioactivity of MOLE. Among the three solvents evaluated, ethanol consistently demonstrated superior antibacterial and antioxidant activity. The ethanolic extract not only completely inhibited MRSA at a lower concentration but also exhibited efficacy comparable to vancomycin, underscoring its potential as a potent natural antimicrobial agent. These findings identify ethanol as the optimal solvent for enriching MOLE with a broad spectrum of bioactive compounds, including flavonoids and phenolic acids, which likely contribute synergistically to its therapeutic effects. Given its potent anti-MRSA and antioxidant properties, the ethanolic MOLE is a strong candidate for development into topical therapeutic formulations, such as wound-healing gels or antiseptic creams for skin infections. More broadly, this work highlights a crucial strategy in combating antimicrobial resistance, optimising the extraction of plant-based compounds to unlock their full potential. The principles demonstrated here can be applied to target other multidrug-resistant pathogens, positioning optimised natural extracts as a vital resource in the development of new antimicrobial agents. Further *in vivo* validation and safety assessments are the essential next steps to translate these promising findings into tangible clinical and pharmaceutical applications.

## Figures and Tables

**TABLE 1:** Bioactive compounds identified in MOLE extracts with antibacterial and antioxidant properties using LC-MS.

Compound	Ethanol extract	Methanol extract	Aqueous extract	Known bioactivity	Classification	Reference
Quercetin-3-galactoside	■■■	□	□	Antioxidant, antimicrobial	Flavonoid glycoside	Nguyen & Bhattacharya (2022); Jing *et al*. (2022)
Quercetin-3-malonyl-glucoside	■	■■■	■■	Antioxidant		
Kaempferol-7-O-glucoside	■■■	□	□	Antioxidant	Flavonoid	Shi *et al*. (2024); Jing *et al*. (2022)
Saponarin	■■■	■■■	■■	Antioxidant, antimicrobial		
Astragalin	■■	■	□	Antioxidant		
Isovitexin	■■	□	□	Antioxidant, antimicrobial	Flavone C-glycoside	Thongphichai *et al*. (2023)
2′-Hydroxygenistein glycoside	□	■■	■	Antioxidant	Isoflavone glycoside	Giner *et al*. (2022)
Chlorogenic acid	□	■■	■■	Antioxidant, antimicrobial	Phenolic acid	Sang *et al*. (2024); Rahmawati *et al*. (2024)
Gallic acid	■	■■	■■■			
Plumieride	■■	■	□	Antioxidant	Iridoid glycoside	Tang *et al*. (2024); Patel (2024)
Kenposide B	■	□	□			
Hydrojuglone glucoside	■■	■■	□	Antioxidant	Naphthoquinone	Giner *et al*. (2022)
Secoisotetrandrine	■	□	□	Antioxidant, antimicrobial	Alkaloid	Zhai *et al*. (2023)
Compound	Ethanol extract	Methanol extract	Aqueous extract	Known bioactivity	Classification	Reference
Orobol 8-C-(6″-acetylglucoside)	■	□	□	Antioxidant	Isoflavone	Siripongvutikorn *et al*. (2024)
Lanceotoxin A	■	□	□	Antimicrobial	Toxin	Kolodziejczyk-Czepas & Stochmal (2017)
Desulfoglucotropeolin	■	□	□	Antimicrobial	Glucosinolate	Abdel-Massih *et al*. (2023)

*Note:* ■■■ = High peak intensity; ■■ = Moderate; ■ = Low; □ = Not detectable
